# Complicated left isolated internal iliac artery aneurysm (IIIAA) in a young man - An unusual presentation in a Tertiary Hospital in Ghana: Case report

**DOI:** 10.1016/j.heliyon.2024.e24039

**Published:** 2024-01-03

**Authors:** Lily P. Wu, Jessica Dei-Asamoa, Benard Ohene-Botwe

**Affiliations:** a Department of Surgery, University of Ghana Medical School, College of Health Sciences, Korle Bu, Accra, Ghana; b Department of Surgery, Korle Bu Teaching Hospital, Accra, Ghana; c Department of Radiography, School of Health & Psychological Sciences, City, University of London, UK; d Department of Radiography, SBAHS, University of Ghana, Accra, Ghana

**Keywords:** Symptomatic DVT, Iliac aneurysm, Young man, Ghana, West Africa

## Abstract

Generally, an aneurysm is a disease of the elderly due to the degenerative aetiological factor. Isolated internal iliac artery aneurysm (IIIAA) is rare, representing 0.3–0.5 % of all intra-abdominal aneurysms. It is a focal dilatation of the internal iliac artery alone with a threshold for surgical intervention set at 8 mm. Herein, we present an unusual presentation of a rare condition of a huge left internal iliac artery aneurysm in a young man with no identifiable risk factor complicated by left ilio-femoral deep vein thrombosis. Even though this is an interesting case study, the lack of facilities to do anaerobic cultures remains a major limitation in our setting.

## 1 Introduction

Iliac artery aneurysm (IAA) is defined as a dilatation of the common, internal or external iliac arteries or various combinations of them, without the involvement of the infrarenal abdominal aorta. Most iliac artery aneurysms are associated with abdominal aortic aneurysms (AAA). Aorto-iliac aneurysms account for 10 % of Abdominal Aortic Aneurysms [1]. Isolated internal iliac artery aneurysm (IIIAA) is rare, representing 0.3–0.5 % of all intra-abdominal aneurysms. It is defined as focal dilatation of the internal iliac artery alone with a threshold for surgical intervention set at 8mm [1,2].

The majority of patients with IAA are elderly aged 65–75 years with male-to-female ratios of between 5–16:1 [[2], [3], [4], [5], [6]].

It is therefore a disease of the aged population. Several risk factors and aetiologies have been described in isolated internal iliac artery aneurysms but in many cases, it is due to degeneration. The other aetiological factors are infection, trauma and arterial wall disorders due to connective tissue diseases like Marfan and Ehlers-Danlos syndrome, fibromuscular dysplasia, Takayasu's arteritis, Kawasaki disease and Behcet's disease. Organisms responsible for the infective causes include Salmonella spp., *Staphylococcus aureus*, *Escherichia coli*. *Pseudomonas* spp., *Klebsiella* spp., Syphillis and Tuberculosis [[7], [8], [9]].

IIIAA is an uncommon cause of DVT [10,11]. We report an uncommon case of a 22-year-old male presenting with a left iliofemoral vein thrombosis due to an IIIAA.

## 2 Case

### 2.1 Patient history

A 22-year-old unemployed male from the eastern region of Ghana was referred to the Vascular Unit of Korle Bu Teaching Hospital (KBTH) with a diagnosis of left leg proximal deep vein thrombosis (DVT) of one-month duration, confirmed by venous duplex scan on the day of admission. There was no preceding history of trauma or symptoms suggestive of syphilis or tuberculosis. The patient was managed on anticoagulation (IV unfractionated heparin 5000 IU 8 hourly) in another hospital for 2 weeks without any improvement. Rather, his condition worsened with progressive swelling and pain in the left leg resulting in his inability to stand, walk or even sit in bed. He has no risk factors for DVT and no family history of DVT.

### 2.2 Physical examination

**Fig. 1:**
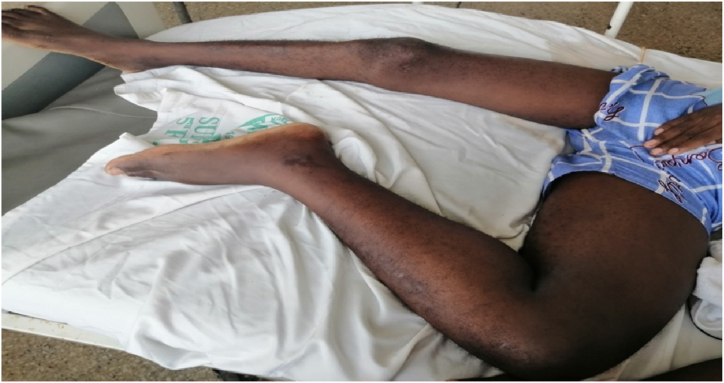
A picture of the patient pre-operative showing limb posture and limb circumference discrepancy.

**Fig. 2:**
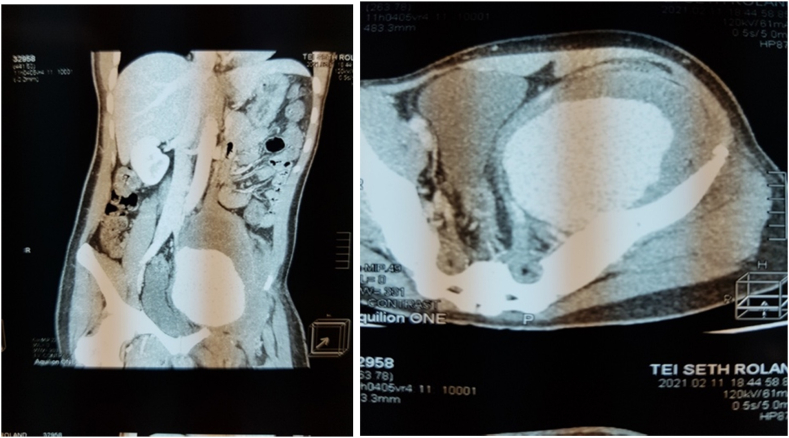
Pre-operative Computerized tomography angiogram (CTA) showing a huge left internal iliac artery aneurysm.

Physical examination revealed a young man with a BMI of 23.2 and no evidence of chronic disease. Blood pressure was 134/92 mmHg and pulse rate was 115 beats/min. He looked acutely unwell with a temperature of 37.9 °C. The left lower limb was held in flexion and external rotation at the hip. It obviously looked bigger than the right one, ankle, calf and mid-thigh circumferences were 23.5cm, 35cm and 48.5cm respectively compared to 20cm, 30cm and 39cm on the right, Fig. 1. He had the full complement of peripheral pulses in both lower limbs. The left lower limb was tender; sensation was intact but motor function was reduced (2/5). A diagnosis of symptomatic ilio-femoral DVT was made. A CT Venogram, Fig. 2, confirmed left ilio-femoral DVT and an 8cm diameter left internal iliac artery aneurysm. Also shown was left hydronephrosis and hydroureter due to ureteric compression by the aneurysm.

### 2.3 Investigations

The various laboratory and radiological tests performed and the corresponding findings/results are presented in Table 1.

**Table 1:** Laboratory and radiological examinations performed and their corresponding results.

Tests	Findings			
FBC	HB 7.9 g/dL (10–14)	WBC 13.86 × 10^9^ (2.50–8.50)	PLT 438 × 10^9^ (150–450)	
BUE, Cr	CR 115.0 (71–133)	UREA 11.2 (2–7)	NA 122 (135–150)	K 5.5 (3.5–5.5)
Hepatitis B and C	Negative			
HIV screen	Negative			
Syphilis screen	Negative			
Blood culture	No bacteria growth			
Sputum for AFBs	Negative			
Chest x-ray	Normal			
ECG	Normal			
Echocardiogram	Normal			
Computerized tomography venogram (CTV)	8 cm left IIAA and ilio-femoral DVT			

### 2.4 Management

**Fig. 3:**
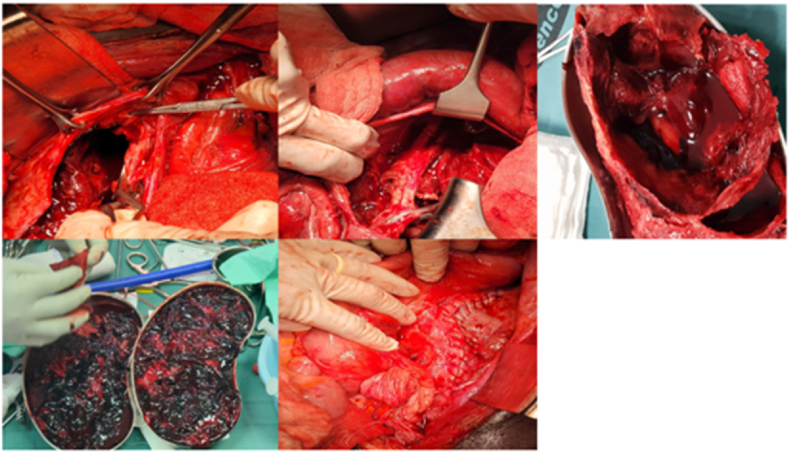
Intra-operative pictures showing the aneurysmal cavity, the evacuated thrombus and the final repair.

The patient was admitted and made comfortable in bed with pressure points padded. He was transfused 4 units of whole blood pre-operative to correct anaemia. Two more units of whole blood and 4 units of fresh frozen plasma were transfused intra-operative. The patient was also given IV amoxiclav 1.2g 12 hourly and IV ciprofloxacin 400mg 12 hourly for 4 days pre-operative and IV antibiotics continued for one week post-operative. IV unfractionated heparin 8000 IU 8 hourly was continued pre-operative. Pain in the left leg was controlled with IV paracetamol 1g 8 hourly and IM pethidine 50 mg 6 hourly pre-operative and post-operative for a total of two weeks. Definitive treatment included *trans*-femoral (via right common femoral artery) insertion of prophylactic IVC filter (optease, Cordis), proximal and distal control were at the right common iliac artery and right external iliac artery respectively. Bolus IV 5000 IU unfractionated heparin was given and activated clotting time (ACT) was 260 seconds prior to proximal and distal control. Stitch ligation of the left internal iliac artery stump off the left common iliac artery was done using prolene 5-0 and primary repair of the left external iliac artery due to iatrogenic injury was also repaired with prolene 6-0. The aneurysmal sac was excised and found to contain both pus and laminar thrombus suggestive of a mycotic origin. Post-evacuation of the intra-aneurysmal thrombus, the large cavity left behind could admit three abdominal sponges and is estimated at 15cm in diameter. The cavity collapsed over 3 weeks period post-operative using a 200ml pneumovac drain set. The pus was sampled for culture and sensitivity but once again no bacteria growth after 5 days. Fig. 3.

### 2.5 Clinical course

Post-operative recovery was uneventful. The length of hospital stay was 20 days. The patient had IV antibiotics for one week post-operative and switched to oral antibiotics for another week. Daily clinical examination for pulses and viability of the lower limbs was done. The patient made good progress with physiotherapy and was able to mobilize with a Zimmer frame prior to discharge home. Haemoglobin prior to discharge was 10.4g/dL, and full blood count and renal function were normal prior to discharge. Pneumovac was removed on postoperative day 10 as the 24-h drainage reduced to 0 ml and the arterial duplex scan showed intact repair and good flow in the left CIA and EIA. Clinically the patient maintained a full complement of peripheral pulses in both lower limbs. He was seen on an outpatient basis for 2 weeks and 3 months post-discharge by which time he was independently ambulant without a walking aid and doing well.

Written informed consent for the case to be published (including images, case history and data) was obtained from the patient.

## 3 Discussion

Improving healthcare delivery requires a robust health policy database. Record keeping and publishing of the changing trend in disease patterns and causes of death in contemporary times are important in achieving this aim. In the Global Burden of Disease Study 2010, non-communicable diseases (NCDs) account for the increasing burden of morbidity and mortality even in modern times accounting for two of every three deaths (34·5 million) worldwide by 2010 [12]. Vascular diseases as part of NCDs are also assuming prominence in the global burden of the disease accounting for one out of every four deaths in 2010 compared with one in five in 1990 [12].

Aneurysm is generally a disease of the aged, above 65 years. This is partly due to the fact that degenerative changes with ageing account for most of its aetiology. Internal Iliac aneurysm (IIA) is a relatively uncommon vascular disease, especially among young people below the age of 50. It is twice as common on the left side compared to the right [6,13]. Bilateral IIA is even rarer [2]. Due to the deep location of the internal iliac artery within the pelvis, the majority of IIIAA cause no symptoms until they either assume a very large size or rupture [2]. Symptoms include pelvic pain which is due to nerve root compression and pressure on neighbouring structures [6,14]. A common clinical presentation is recurrent urinary tract infections due to ureteric compression which can lead to hydronephrosis. Deep vein thrombosis and arteriovenous fistulae have also been described [10,11,15]. In a study by Laine et al. [6], the mean diameter of ruptured IIIAA was 6.8 cm, 4.4 times (range, 0.7–11.4 times) the diameter of the contralateral IIA. The timely diagnosis and prompt surgical treatment of a left IIIAA complicated by progressive left proximal DVT despite adequate medical treatment is worth publishing to raise awareness among health professionals.

An isolated internal iliac artery aneurysm is rare. Symptoms are late in onset and non-specific. It is even rarer to have this disease occur in people below the age of 50 years. Our patient is only 22 years old with IIIA complicated by hydronephrosis and proximal DVT. At presentation, the combination of fever, a short duration of symptoms, and a large aneurysm diameter suggests a rapid increase in size. Coupled with the presence of pus in the aneurysmal sac, it is likely to be of an infective cause (mycotic aneurysm). A high index of suspicion is required in making a prompt diagnosis and appropriate treatment.

## 4 Conclusion

The rarity of IIIAA especially at a young age, the rapidity with which the aneurysmal sac increased in size and the presence of fever and pus in the sac point to a mycotic origin. A high index of suspicion must be exercised to arrive at the diagnosis of proximal DVT as a complication supported by the non-response to therapeutic anti-coagulation therapy. This paper when published may raise awareness among clinicians to better manage such patients promptly.

## Data availability statement

The raw/processed data required to reproduce the above findings cannot be shared at this time as the data also forms part of an ongoing study.

## Funding

None.

## Ethical approval

Ethical approval for this study was waived but written informed consent for the case to be published (including images, case history and data) was obtained from the patient.

## CRediT authorship contribution statement

**Lily P. Wu:** Writing - review & editing, Writing - original draft, Visualization, Validation, Supervision, Software, Resources, Project administration, Methodology, Investigation, Funding acquisition, Formal analysis, Data curation, Conceptualization. **Jessica Dei-Asamoa:** Writing - review & editing, Writing - original draft, Visualization, Validation, Resources, Project administration, Methodology, Investigation, Formal analysis, Conceptualization. **Benard Ohene-Botwe:** Writing - review & editing, Writing - original draft, Validation, Resources, Methodology.

## Declaration of competing interest

The authors declare that they have no known competing financial interests or personal relationships that could have appeared to influence the work reported in this paper.
